# Up to 5-year retention of abatacept in Belgian patients with moderate-to-severe rheumatoid arthritis: a sub-analysis of the international, observational ACTION study

**DOI:** 10.1007/s00296-020-04619-z

**Published:** 2020-06-17

**Authors:** R. Westhovens, S. E. Connolly, J. Margaux, M. Vanden Berghe, M. Maertens, M. Van den Berghe, Y. Elbez, M. Chartier, F. Baeke, S. Robert, M. Malaise

**Affiliations:** 1 Department of Development and Regeneration, Skeletal Biology and Engineering Research Center Leuven, University Hospitals Leuven, Herestraat 49, 3000 Leuven, Belgium; 2 Bristol-Myers Squibb, Princeton, NJ USA; 3 Rheumatology and Physical Medicine Department, Erasme Hospital, Brussels, Belgium; 4 Grand Hôpital de Charleroi, Charleroi, Belgium; 5 AZ Damiaan, Oostende, Belgium; 6 ASZ Aalst, Wetteren, Belgium; 7 Excelya, Boulogne-Billancourt, France; 8 Bristol-Myers Squibb, Rueil Malmaison, France; 9 Bristol-Myers Squibb, Braine-l’Alleud, Belgium; 10 CHU Sart Tilman, Liège, Belgium

**Keywords:** Abatacept, Effectiveness, Retention, Long-term outcomes, Rheumatoid arthritis

## Abstract

Favorable efficacy and safety profiles have been demonstrated for abatacept in patients with rheumatoid arthritis (RA) in randomized controlled trials, but these data require validation during long-term follow-ups in routine clinical practice. This study explored long-term safety and retention rates in RA patients treated with intravenous abatacept in the Belgian cohort of the international AbataCepT In rOutiNe clinical practice (ACTION) study (NCT02109666). This non-interventional, observational, longitudinal study included Belgian patients aged ≥ 18 years with moderate-to-severe RA who started intravenous abatacept treatment as first- or second/further-line biologic therapy in routine clinical practice. Between October 2010 and December 2012, 141 patients were enrolled in this cohort, of whom 135 evaluable patients (6 biologic-naïve; 129 previously exposed to ≥ 1 prior biologic disease modifying anti-rheumatic drugs) were eligible for the descriptive analysis; 131/135 were included in the effectiveness analysis. Mean disease duration was 10.5 years (standard deviation 9.7) before abatacept initiation. RA patients presented with high disease activity and comorbidity rate, having failed multiple previous treatment options. In this cohort, the 5-year abatacept retention rate was 34% (95% confidence interval, 23−45%) per protocol, and 51% (95% confidence interval, 40−61%) when temporary discontinuations of abatacept > 84 days (*n* = 24) were not considered as treatment discontinuations. After 5 years of abatacept treatment, clinical outcomes were favorable [good/moderate European League Against Rheumatism (EULAR) responses in 91.7% patients]. No new safety signals were detected for abatacept in routine clinical practice. In this difficult-to-treat Belgian RA population, high retention rates, good clinical outcomes and favorable safety profile were observed with abatacept.

**Electronic supplementary material:**

The online version of this article (10.1007/s00296-020-04619-z) contains supplementary material, which is available to authorized users.

## Introduction

Rheumatoid arthritis (RA) is an autoimmune disease characterized by uncontrolled inflammation of the synovial tissue in the joints. Following its natural course and when left untreated, this chronic condition usually leads to progressive joint damage, functional disability, impaired quality of life [1–3] and shortened life expectancy [4, 5]. Over the past years, novel treatment strategies and pharmacological treatment options have been developed, aiming to reach and maintain disease remission in order to prevent worsening of structural damage, disability and allow patients to better participate in daily life activities.

First-line treatment usually includes conventional synthetic disease-modifying anti-rheumatic drugs (csDMARDs), among which methotrexate (MTX) is the most widely used, often in combination with temporary glucocorticoids. When treatment with csDMARDs fails, biological DMARDs may be introduced [6].

Among the array of available biologicals, abatacept, a fully humanized cytotoxic T-lymphocyte associated protein 4 (CTLA4)-Ig fusion protein, counteracts the progression of joint damage by interfering with CD28-CD80/86 T cell co-stimulation therewith alleviating the autoimmune inflammatory reaction [7] and by reducing cluster of differentiation 80/86 (CD80/86)-driven osteoclast formation [8, 9]. Abatacept is available in subcutaneous (SC) and intravenous (IV) formulations. Favorable efficacy and safety profiles have been demonstrated for abatacept in randomized controlled trials (RCTs), both in biologic-naïve and biologic-experienced RA patients [10–13]. In the long term, consistent safety and sustained efficacy were shown for abatacept over 7 years in an extension trial of MTX-inadequate responders with established RA [14]. An acceptable safety profile was also documented from the total clinical trial program including the evaluation of safety data from 8 trials of the IV abatacept clinical development program with abatacept exposure up to 8 years [15].

Treatment responses in routine clinical practice may however differ from those observed in clinical trials, as the patient population is not subject to strict inclusion/exclusion criteria and thus more diverse. For instance, overall, lower treatment response rates to tumor necrosis factor (TNF)-blocking agents have been reported in RA routine clinical practice when compared to RCTs [16]. Therefore, long-term efficacy and safety of treatment in a chronic disease such as RA require validation in routine medical practice. The AbataCepT In rOutiNe clinical practice (ACTION; ClinicalTrials.gov identifier: NCT02109666) study provided crucial data on the retention of abatacept and prognostic factors of retention in patients with RA in routine clinical practice across Europe and Canada [17–19].

Determinants of response stability and treatment discontinuation are essential to guide physicians in decision making of individualized treatment regimens [17]. As concomitant treatments, treatment histories, and demographic characteristics of RA patients treated with abatacept may vary substantially among countries due to differences in terms of availability and access to biological agents, reimbursement policies (time to reimbursement and reimbursement criteria, such as the minimum level of disease activity [20]) and standards of care according to the social system (e.g. glucocorticoid use) [21], the local perspective of retention rates and prognostic factors are assessed in this Belgian cohort of the ACTION study.

## Materials and methods

### Study design and study population

This non-interventional, observational, longitudinal study was conducted at 16 different centers in Belgium as part of the international ACTION study [17]. It included adult (≥ 18 years of age) moderate-to-severe RA patients enrolled between October 2010 and December 2012, who started treatment with IV abatacept as first- or second/further-line biologic therapy in routine clinical practice. Patients participating in any interventional clinical trial on RA were not included in the study.

**Fig. 1:**
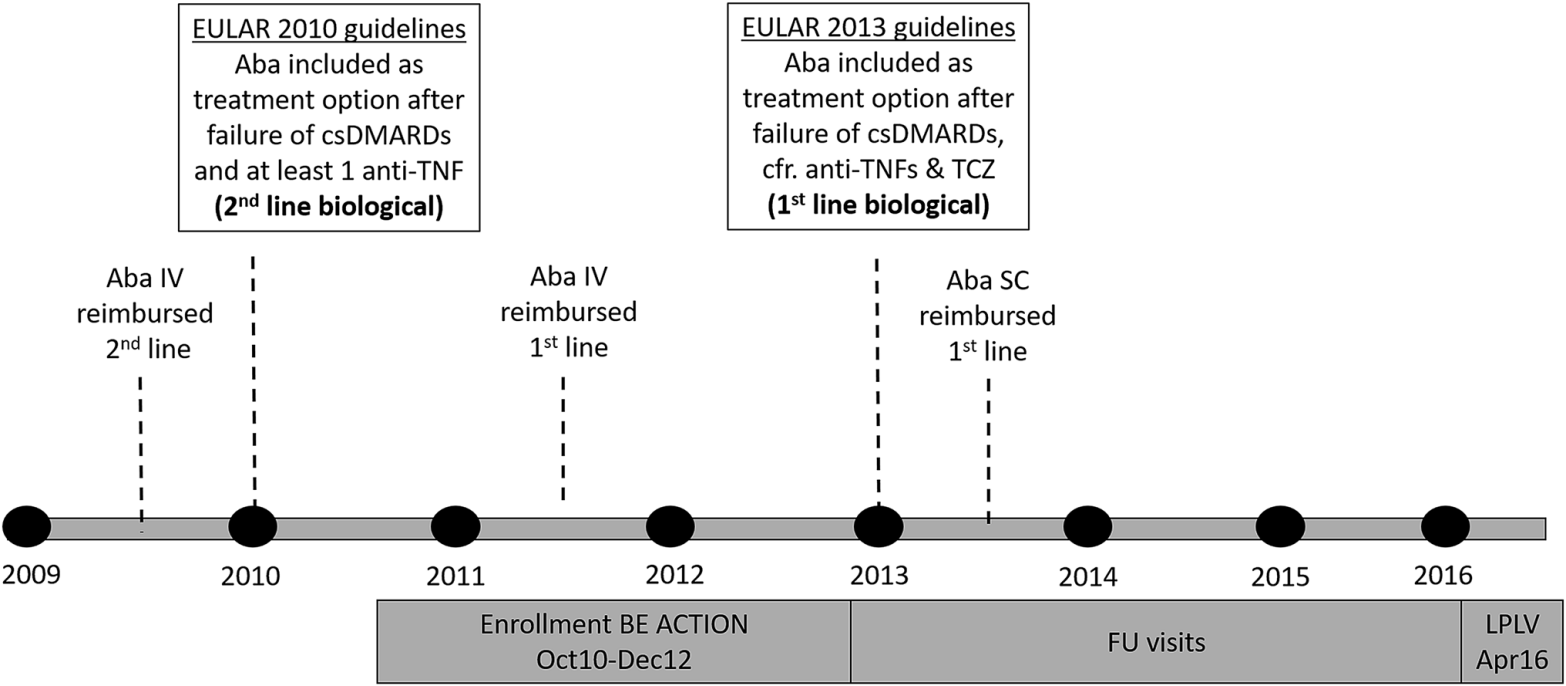
Reimbursement timeline for abatacept in Belgium. *Aba* abatacept, *ACTION* AbataCepT In rOutiNe clinical practice, *BE* Belgium, *csDMARDs* conventional synthetic disease-modifying anti-rheumatic drugs, *EULAR* European League Against Rheumatism, *FU* follow-up, *IV* intravenous, *LPLV* last patient last visit, *SC* subcutaneous, *TCZ* tocilizumab, *TNF* tumor necrosis factor

According to the Belgian reimbursement criteria for abatacept applicable at the time of study initiation, patients with a 28-joint Disease Activity Score using C-reactive protein (DAS28[CRP]) > 3.7 and without contraindications as judged by the treating rheumatologist received abatacept IV as second-line biological after failure of at least 2 csDMARDs (including MTX) and at least one anti-TNF agent. In 2011, IV abatacept became also reimbursed as first-line biological treatment (after failure of 2 csDMARDs including MTX) and patients receiving abatacept as first-line biologic DMARD were allowed to enroll in the study. In 2013, SC abatacept was introduced and reimbursed in Belgium, allowing study patients to switch from IV to SC abatacept administration (Fig. 1). For patients treated with abatacept as first-line biological, follow-up was approximately every 3 months for maximum 2 years. Other patients had a follow-up of maximum 3−5 years. Of note, this observational study did not interfere with the physician’s routine clinical practice, and the decision to treat patients with abatacept was made before their enrolment in the study.

The global study was conducted in accordance with the Declaration of Helsinki, International Conference on Harmonization's Guideline for Good Clinical Practice and Good Epidemiological Practice, and with the approval of the Central Ethics Committee (Ethik-Kommission der Bayerischen Landesärztekammer; IM101151) on November 1, 2008. The Belgian part of the study has been approved by a Central Ethics Committee (Commissie Medische Ethiek of the Universitaire Ziekenhuizen K.U.Leuven) on October 4, 2010. All patients provided written informed consent.

### Assessments

The primary study objective was to estimate the retention rate (consecutive time on treatment) of abatacept in Belgian RA patients treated over 24 months as first-line treatment or over 36−60 months as second- or further treatment line in routine clinical practice. The secondary study objective was to identify major determinants of treatment discontinuation (including temporary discontinuation and feasibility of treatment restart) in Belgian RA patients treated with abatacept.

Clinical characteristics and effectiveness were reported for patients with data available at baseline, assessed within 8 days after the first abatacept infusion. Patients who had their clinical assessment more than 8 days after their first abatacept infusion were not included in the effectiveness analysis. Disease activity was evaluated using the 28-joint disease activity score (DAS28), based either on erythrocyte sedimentation rate (ESR) or C-reactive protein (CRP) [22, 23] according to physician’s choice, and clinical disease activity index (CDAI) [24]. Data were either collected retrospectively at baseline (socio-demographics, disease history and characteristics, prior RA treatments such as biologic or csDMARDs, and other concomitant medication) or prospectively (clinical and patient-reported outcomes) at baseline and during follow-up with approximate 3-month intervals (at the physician’s discretion).

Safety was evaluated in accordance with local regulations and registered with the drug manufacturer’s global pharmacovigilance department. Related treatment-emergent adverse events (AEs) were assessed by the treating physician and reported to the pharmacovigilance department. The relationship between the study drug and serious AE (SAE) was judged by the treating physician. Safety was presented for the entire enrolled population, regardless of prior or concomitant treatment.

### Statistical analysis

Baseline demographic data and disease characteristics were reported using descriptive statistics including sample size, mean [standard deviation (SD)] for continuous variables or frequency (%) for categorical variables. Descriptive analyses were presented for all evaluable patients.

Abatacept retention rates with corresponding 95% confidence intervals (CIs) were calculated based on the number of events (treatment discontinuations) estimated by Kaplan–Meier analysis. Retention was defined as consecutive time on treatment. Switches from IV to SC abatacept during the study were not considered as events. Temporary abatacept discontinuations were defined as periods of more than 84 days without abatacept doses in participants who restarted subsequently. Temporary discontinuations were deemed necessary by the responsible physician mainly in case of interfering infections or surgery. Per protocol, these temporary abatacept discontinuations were considered treatment discontinuations (they were included in the number of events in the Kaplan–Meier analysis). A separate analysis was performed where these temporary discontinuations were not considered treatment discontinuations (they were excluded from the number of events in the Kaplan–Meier analysis).

Potential explanatory variables of abatacept discontinuation were identified using univariate analysis with a Cox-proportional hazard model for clustered data to account for dependence of data from patients enrolled by the same investigator. Clinically relevant variables, known risk factors and prognostic factors with *p* < 0.20 in the univariate analysis were entered into a multivariate Cox proportional hazards regression model. Co-linearity between potential prognostic factors was assessed. Two categorical variables were considered as colinear if the Chi-Square test *p* value was < 0.05 and V-Cramer > 0.5. Results were presented as hazard ratios (HRs) with corresponding 95% CI and *p* values. Multivariate analyses were performed considering clinical disease activity both as continuous and categorical value (< median, > median). No imputation for missing data was used.

Frequencies of AEs were summarized descriptively.

## Results

### Study population

**Fig. 2:**
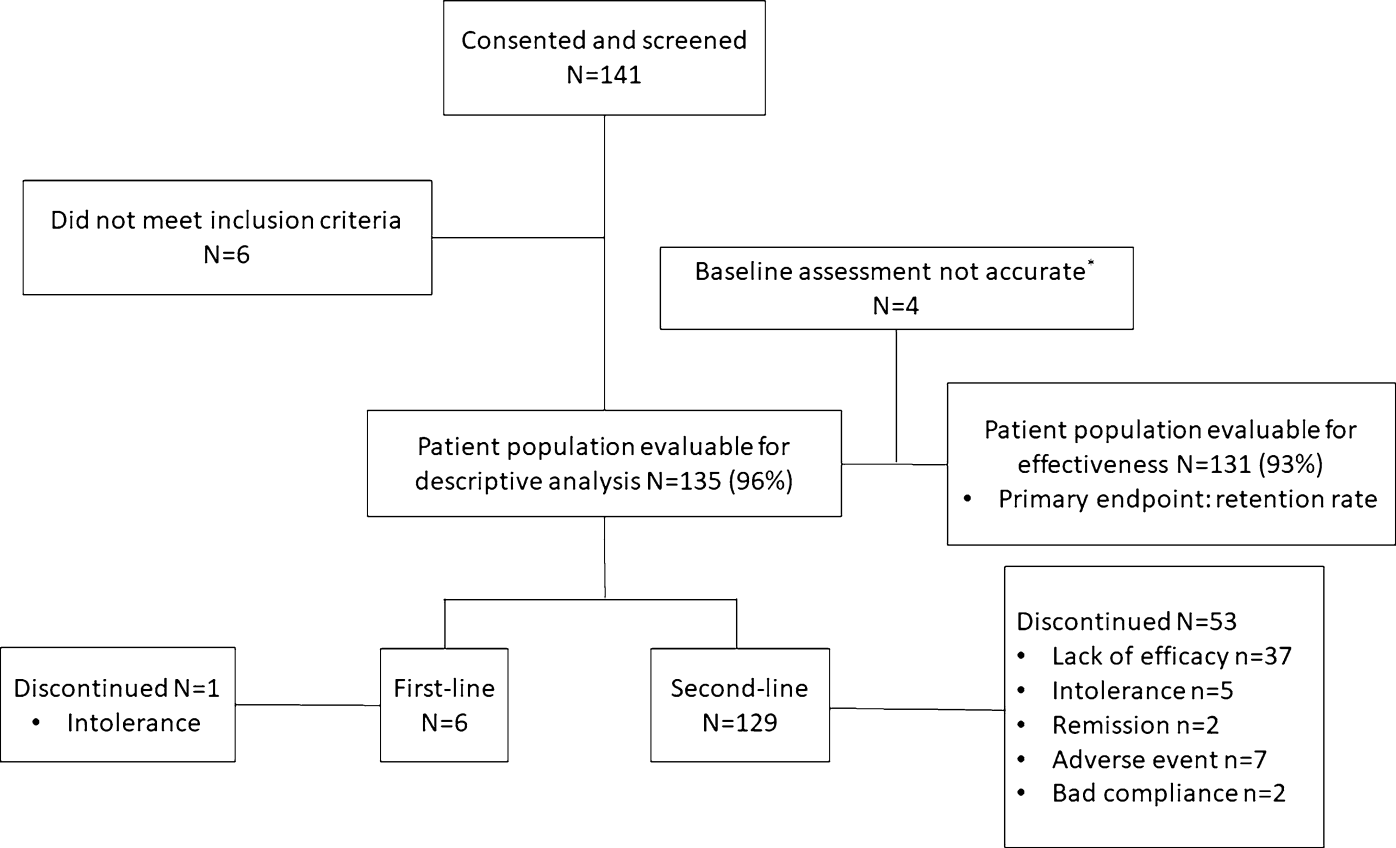
Patient disposition. *N* total number of patients per category, *n* number of patients per category ^*^Clinical assessment was performed not later than 8 days after the first abatacept infusion

Between October 2010 and December 2012, 141 patients were enrolled (consented and screened) in this cohort, of whom 135 evaluable patients (6 biologic-naïve and 129 previously exposed to ≥ 1 prior biologic) were eligible for the descriptive analysis (Fig. 2). Of these, 97% (131/135) were included in the effectiveness analysis (4 patients were excluded as their baseline clinical assessment took place more than 8 days after their first abatacept infusion). The mean number of DMARDs received prior to enrolment in the study was 2.15 (Table 1). Only 13 (9.6%) patients received > 3 DMARDs prior to treatment with abatacept. Overall, 93.3% (126/135) patients received anti-TNF treatment(s) before abatacept initiation: of these, 58.7% (74/126) received 1 prior anti-TNF agent and 41.3% (52/126) received 2 or more prior anti-TNF agents. Moreover, 27.4% (37/135) patients received biologic treatment(s) not based on anti-TNF agents prior to abatacept treatment. Reasons for discontinuation of the last biologic treatment before study enrolment were reported for 84 patients and included primary inefficacy 31% (26/84), secondary inefficacy 53.6% (45/84), safety and tolerability 13.1% (11/84), and other unspecified reasons 2.4% (2/84).

**Table 1:** Baseline demographics and clinical characteristics

Characteristics	Value
Age, years	57.04 (11.75)
Female, *n* (%)	104 (77.0)
BMI, kg/m^2^	26.63 (6.04)
BMI category, *n* (%)	
< 25 kg/m^2^	70 (51.9)
≥ 25 kg/m^2^ and < 30 kg/m^2^	41 (30.4)
≥ 30 kg/m^2^ and < 35 kg/m^2^	14 (10.4)
≥ 35 kg/m^2^	10 (7.4)
RA duration in years	10.54 (9.66)
ESR (mL)	24.88 (22.94)
CRP (mg/L)	10.68 (18.84)
TJC28	9.82 (6.57)
SJC28	5.81 (5.17)
DAS28 (ESR)	5.21 (1.02)
DAS28 (CRP)	4.72 (1.09)
CDAI	28.53 (11.11)
SDAI	29.91 (11.88)
PtGA, VAS 100 mm	65.30 (20.95)
HAQ-DI	1.23 (0.65)
RF positive, *n*/*N* (%)	79/103 (76.7)
Anti-CCP positive, *n*/*N* (%)	63/88 (71.6)
Presence of cardiovascular risk factors, *n* (%)	56 (41.8)
≥ 1 Co-morbidity, *n* (%)	67 (49.6)
Number of prior non-biologic DMARDs	2.15 (1.05)
Number of prior anti-TNFs	1.44 (0.81)
Concomitant treatment, *n* (%)	
Monotherapy	34 (25.2)
MTX only	79 (58.5)
MTX + other csDMARD	4 (3.0)
Other csDMARD only	18 (13.3)
GC use, *n* (%)	
No GC	23 (17.0)
GC introduced at abatacept initiation	14 (10.4)
Continuous use of GC	70 (51.9)
Stop GC at abatacept initiation	28 (20.7)

Data were aggregated for 1st (*n* = 6) and 2nd/further treatment lines (*n* = 129). Data are mean (SD) unless indicated otherwise

*BMI* body mass index, *CCP* cyclic citrullinated protein antibody, *CDAI* clinical disease activity index, *CRP* C-reactive protein, *DAS28* 28-joint disease activity score, *csDMARD* conventional synthetic disease-modifying anti-rheumatic drug, *ESR* erythrocyte sedimentation rate, *GC* glucocorticoid, *HAQ-DI* health assessment questionnaire disability index, *MTX* methotrexate, *N* number of patients for which the results are known by the physician, *n (%)* number (percentage) of patients in each category, *PtGA* patient global assessment of disease activity, *RA* rheumatoid arthritis, *RF* rheumatoid factor, *SDAI* simplified disease activity index, *SJC* swollen joint count, *TJC*, tender joint count, *TNF* tumor necrosis factor, *VAS* visual analog scale

During the study, 54 patients discontinued treatment and 37 patients switched to SC abatacept administration. Baseline characteristics of the patients who discontinued treatment due to lack of efficacy are analyzed in supplementary Table S1. Ten patients were lost to follow-up.

The mean age of patients initiating abatacept was 57 years, the majority (77.0%) were females and 51.9% had a body mass index (BMI) < 25 kg/m^2^. Overall, patients had a mean disease duration of 10.5 years (SD, 9.7) before abatacept initiation and showed a DAS28 (CRP) of 4.7, a mean CDAI of 28.5 and a mean simplified disease activity index (SDAI) of 29.9 (Table 1).

Percentages of patients presenting with risk factors of disease progression were 71.6% for anti-cyclic citrullinated peptide (CCP) antibody positive (*n*/*N* = 63/88) and 76.7% for rheumatoid factor positive (*n*/*N* = 79/103) (Table 1).

Approximately half of the patients (49.6%, *n* = 67) presented at least one comorbidity at enrolment (Table 1). The most commonly reported comorbidities were endocrine metabolic disorders (20.9%, *n* = 24), respiratory disease (20.0%, *n* = 23), cardiovascular and/or cerebrovascular disease (18.3%, *n* = 21). Other co-morbidities reported at baseline included infections and infestations (6.1%, *n* = 7), hepatic disease (5.2%, *n* = 6), renal disorders (4.3%, *n* = 5), and neoplasms in the past (6.1%, *n* = 7).

On average, patients received two non-biologic csDMARDs prior to enrolment in the study and one anti-TNF agent (Table 1). Twenty-five percent of patients initiated abatacept as monotherapy, whereas the majority (61.5%) initiated abatacept in combination with MTX (58.5% MTX alone and 3.0% MTX plus another csDMARD). Most patients (52%) received glucocorticoids prior to abatacept initiation and continued these upon abatacept initiation. Ten percent of patients started glucocorticoids, whereas 21% of patients discontinued glucocorticoid treatment at abatacept introduction (Table 1).

### Five-year retention and clinical outcomes

**Fig. 3:**
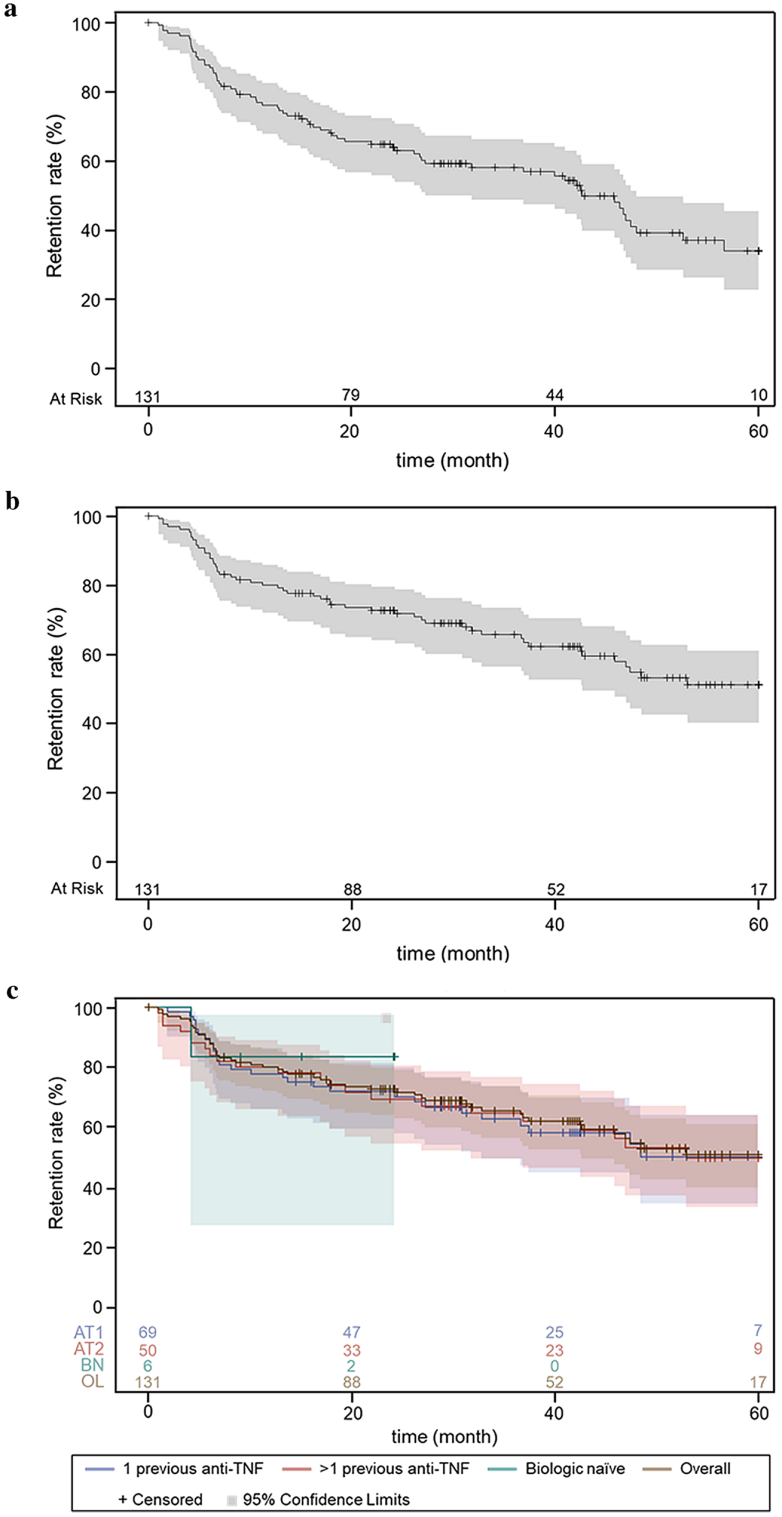
Five-year abatacept retention rates **a** per protocol (i.e. considering temporary discontinuations as treatment discontinuations), **b** when temporary discontinuations were not considered as treatment discontinuations, and **c** in overall population (OL), bio-naïve patients (BN), patients receiving 1 previous anti-TNF (AT1) or more than 1 previous anti-TNF (AT2) before first abatacept infusion

The crude retention rates were 76% (95% CI 68−83%) at 12 months, 64% (95% CI 55−72%) at 24 months and 34% (95% CI 23−45%) at 60 months in the per-protocol analysis (Fig. 3a). When temporary discontinuations of abatacept were not considered treatment discontinuations, retention rates were 80% (95% CI 72−86%) at 12 months, 73% (95% CI 64−80%) at 24 months and 51% (95% CI 40−61%) at 60 months (Fig. 3b). Patient retention rates estimated by Kaplan–Meier analysis over 60 months by prior exposure to anti-TNFs are illustrated in Fig. 3c. The 12-month retention rate was 83% (95% CI 27–97%) in biologic-naïve patients, 77% (95% CI 66–85%) in patients who received only 1 anti-TNF before abatacept initiation and 81% (95% CI 67–90%) in patients who received more than 1 anti-TNF before abatacept initiation. At 24 months, the retention rate was 83% (95% CI 27–97%) in biologic-naïve patients, 71% (95% CI 59–80%) in patients receiving only 1 anti-TNF and 71% (95% CI 55–81%) in patients receiving ≥ 2 anti-TNF before abatacept initiation. The overall crude retention rate at 60 months was 47% (95% CI 30–62%) in patients with ≥ 2 previous biologic failures.

Over 5 years, the most common reasons for discontinuation of abatacept (*n* = 54) were lack of efficacy (*n* = 37) and intolerance and safety (*n* = 13) (Fig. 2). Potential explanatory variables of abatacept discontinuation were investigated using univariate analysis. In total, three variables were retained from the univariate analysis and introduced in the multivariate model. Those factors were reason for discontinuation of last biological agent (*p* = 0.0233), clinical disease activity (*p* = 0.0779) and cardiovascular comorbidity (*p* = 0.1960). No significant factors were retained from the multivariate analysis; only a tendency for less likely discontinuation of abatacept was seen (*p* < 0.10, not significant) in patients with higher CDAI at baseline (data not shown). Patients who discontinued abatacept treatment due to lack of efficacy had a significantly shorter disease duration, higher CRP concentrations and a higher number of prior DMARDs at baseline as compared to the rest of the study population (Supplementary Table S1). At the time of discontinuation, these patients had a mean DAS28 (CRP) ± SD of 3.82 ± 1.31 and a DAS28 (ESR) ± SD of 3.75 ± 1.34.

**Fig. 4:**
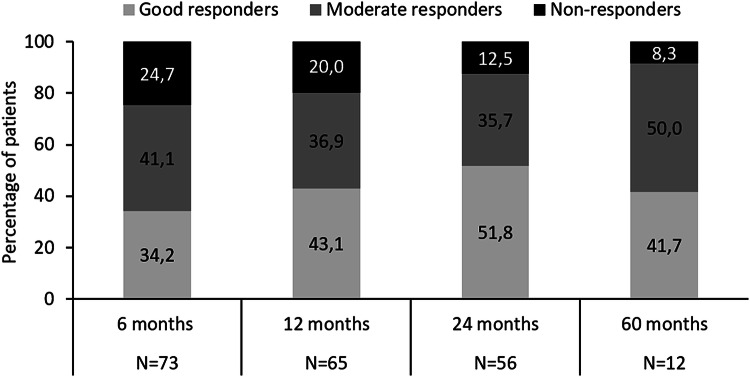
European League Against Rheumatism (EULAR) response over 5 years of abatacept treatment. *EULAR* European League Against Rheumatism, *N* number of patients with available results per category

After 6 months, the proportion of patients obtaining good/moderate European League Against Rheumatism (EULAR) response was 75.3% (*n*/*N* = 55/73) and this proportion further increased over time. Good/moderate EULAR responses were reached for 80% (52/65) of patients at 12 months, 87.5% (49/56) of patients at 24 months and 91.7% of patients (11/12) at 60 months (Fig. 4).

### Temporary discontinuation of abatacept

**Fig. 5:**
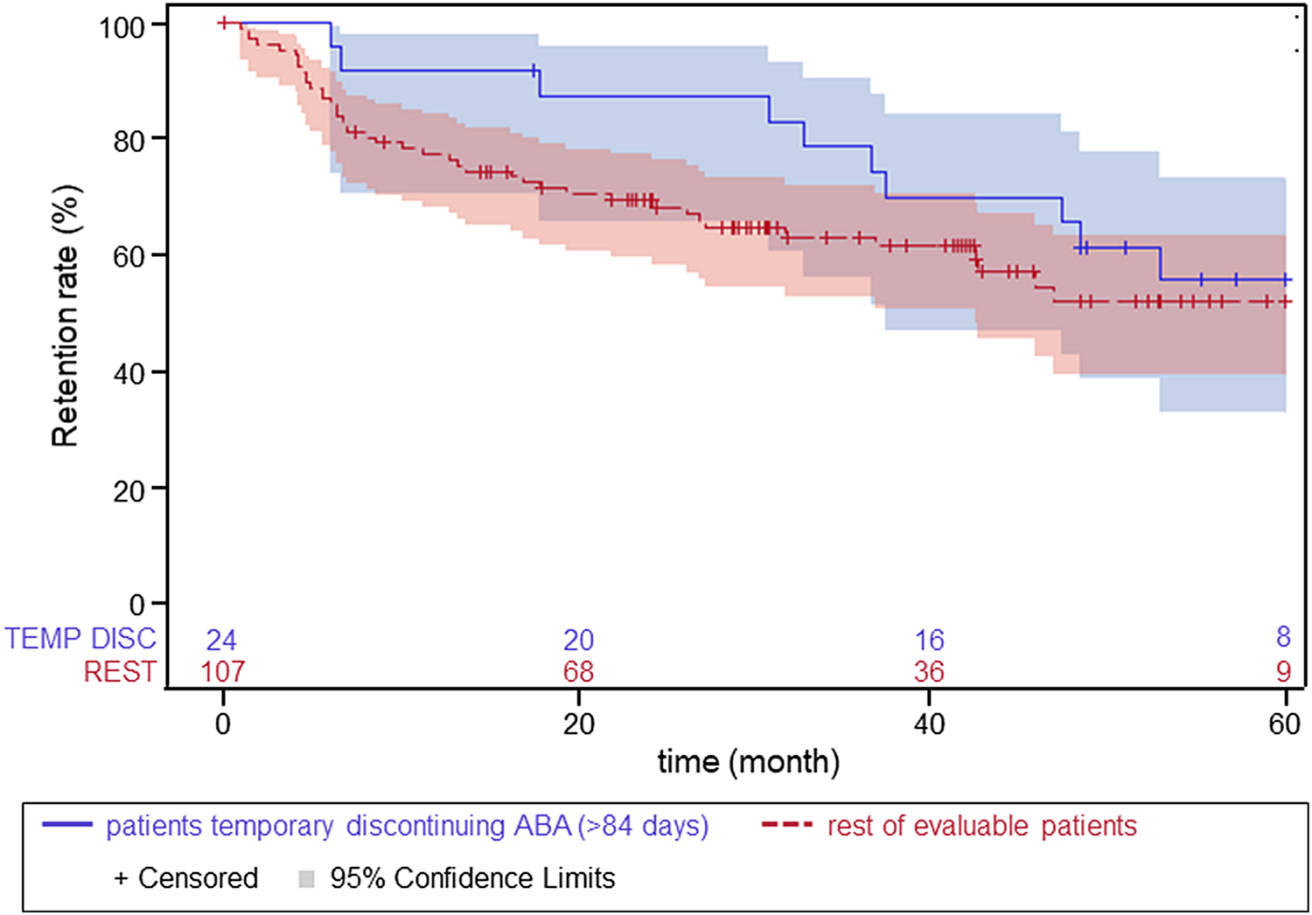
Impact of temporary discontinuation on patient retention rate. Red line indicates patients who temporary discontinued abatacept and blue line indicates the rest of the study population. *ABA* abatacept

During the study, 24 patients interrupted abatacept treatment for > 84 days, after which they restarted treatment (temporary discontinuation). In this group, the DAS28 (CRP) before discontinuation and at restart was stable (3.4 [SD, 1.06] versus 3.6 [SD, 1.08], respectively [*n* = 15]). Patients who temporary discontinued abatacept treatment did not negatively impact the retention in this cohort (Fig. 5).

### Safety

For 40 out of 135 patients, one or more AEs were reported during the study. Overall, 24 cases of AEs and 36 cases of SAEs were reported in these 40 patients. Seven (5.2%) patients discontinued abatacept treatment due to an SAE, and four (3.0%) patients due to an AE. Out of the 22 infections reported, 10 were categorized as serious. Two participants died (necrotizing pancreatitis and complications of bronchiectasis) possibly (not probably) related to the treatment as assessed by the investigator. Overall no new safety signals were detected for abatacept in routine clinical practice.

## Discussion

The Belgian ACTION cohort represents a difficult-to-treat patient population with moderate-to-severe RA, several risk factors for disease progression and multiple co-morbidities and refractoriness to several treatment options before exposure to abatacept. Despite this profile, the 5-year abatacept retention rate in this cohort was 34% (per protocol) and 51% when temporary discontinuations of abatacept > 84 days (*n* = 24) were not considered as treatment discontinuations. In addition, good clinical outcomes (good/moderate EULAR responses in 91.7% of patients after 5 years) could be demonstrated for Belgian RA patients treated with abatacept in routine clinical practice. Temporary discontinuation of abatacept did not have major clinical consequences, which might be different from anti-cytokine therapies where tapering is associated with a number of flares [25]. The favorable safety profile of abatacept was consistent with what has been reported in RCTs and in clinical practice [15, 26].

The efficacy of abatacept observed in the Belgian ACTION cohort is consistent with recent French registry data [27]. In the Orencia and Rheumatoid Arthritis registry, retention rates at 6 months ranged from 58.5 to 76.0% depending on whether abatacept was initiated as monotherapy or in combination with csDMARDs [27]. With its robust design and the long-term (5-year) follow-up window, the ACTION study further complements this information obtained from registries. However, in the global ACTION study, abatacept retention rates varied between countries (with highest retention rates reported for the Belgian cohort [28]), an aspect that has also been observed in a recent publication on abatacept retention in nine European countries [29]. Concomitant treatments, treatment histories, demographic characteristics and reimbursement policies may differ substantially between countries, thus, stressing the need for a local perspective.

Contextualizing the Belgian ACTION results within broader national data is of interest; however, comparison to Belgian cohorts of other biologicals is complicated by differences in study design [30–32]. For golimumab, baseline disease activity in the Belgian population of the GO-MORE trial was lower and 6-month remission rates were higher than in the rest of the world (DAS28 [ESR] 43.1% versus 23.2%; *p* < 0.0001 and SDAI 22.0% versus 13.8%; *p* = 0.01) [30]. For infliximab, sustained clinical benefit could be demonstrated over a 7-year period of time [32]. Mean DAS for patients still on treatment was 3.0 (standard error [SE] 0.1) at year 4 and remained at the same level until year 7 (3.0 [SE 0.1]) [32]. Of note, a recent retrospective study conducted at seven centers in Japan demonstrated that abatacept had the highest overall retention rate and the lowest discontinuation rate in clinical practice, based on toxic AEs among seven biologics (tocilizumab, etanercept, infliximab, abatacept, adalimumab, golimumab, and certolizumab pegol) for RA [33].

Recent data from the AGREE (Abatacept trial to Gauge Remission and joint damage progression in MTX-naïve patients with Early Erosive rheumatoid arthritis) trial indicate that timely induction of abatacept in combination with MTX may be followed by dose reduction in patients with early RA as remission is sustained [34]. Similarly, remission in patients with early RA could be maintained following reduction or withdrawal of the TNF inhibitor, etanercept [35]. In addition to these findings, the data on temporary discontinuation in the Belgian ACTION cohort are reassuring as they demonstrate that, even in a refractory patient population, treatment interruptions are not associated with major clinical consequences. According to the Belgian reimbursement criteria, switching between biological DMARDs is justified if the patient reaches a DAS28 score of 3.7 or above. In this cohort, patients who discontinued abatacept due to inefficacy presented a mean DAS28 > 3.8, indicating that the patients were indeed not well controlled and that physicians were following the guidelines (DAS28 ≥ 3.7). Patients who discontinued abatacept due to inefficacy in this cohort presented significantly higher number of prior csDMARDs, higher CRP level and shorter disease duration.

Most of the patients enrolled (95.6%) in the Belgian ACTION cohort received abatacept as second- or further line treatment, in concordance with the EULAR 2010 recommendations and reimbursement policies of abatacept applicable until 2011 in Belgium. Whereas abatacept is reimbursed as first-line treatment in Belgium since 2011, EULAR guidelines were only adapted in 2013 and served as the main treatment guidance for physicians, explaining the limited number of patients receiving first-line abatacept included in this study. While abatacept retention rates at 24 months were higher in earlier versus later lines in the global study [17], the number of patients receiving first-line abatacept was too small in the Belgian cohort to draw any conclusions on first- versus later lines. In 2013, during the follow-up period of the study, SC abatacept became available on the Belgian market, allowing patients in routine practice to opt for a more convenient formulation. IV and SC abatacept administration have shown equal efficacy in RA patients [36]. Thus, switching from IV to SC administration could be considered indicative for a patient/physician’s preference for SC formulation. In this cohort reflecting routine clinical care, 35 patients switched from IV to SC administration and they did not show a significant change in DAS28.

Despite the EULAR recommendations suggesting that biological DMARDs should be used in combination with MTX in RA treatment [37], in general up to one-third of patients with RA are treated with monotherapy [38]. It has been demonstrated that efficacy and safety of abatacept initiated alone or in combination with csDMARDs is similar and that abatacept monotherapy may thus serve as an alternative when csDMARDs are not adequate [39]. A different study showed that adding MTX to abatacept did not further improve treatment response in patients with RA after non-TNF inhibitor inadequate response [40]. In the Belgian ACTION cohort, the majority of patients initiated abatacept with MTX and one quarter received abatacept as monotherapy. More than half of the patients in the Belgian ACTION cohort had already received glucocorticoids before abatacept initiation and continued the treatment. However, 10% of the patients initiated glucocorticoid treatment in combination with abatacept and 21% discontinued glucocorticoid use upon abatacept initiation. While information on concomitant use of glucocorticoids and biological agents is scarce, studies have provided evidence that treatment with biologic agents may reduce the use of glucocorticoids [41–43].

Predictors of retention differ between the international ACTION study and the Belgian ACTION cohort. While in the international ACTION study patients had a significantly lower risk of abatacept discontinuation if they were anti-CCP positive, had failed < 2 anti-TNF agents, or had a cardiovascular comorbidity at abatacept initiation [44], patients in the Belgium ACTION cohort tended only to discontinue abatacept less likely (*p* < 0.10, not significant) with higher CDAI. Using two large United States insurance claims databases, it was recently shown that abatacept is associated with a 20% reduced risk of cardiovascular diseases when compared to TNF inhibitor [45].

The following limitations inherent to the design of non-randomized trials conducted in routine clinical practice should be noted: referral and channeling bias, lack of an active comparator and loss of patient follow-up (*n* = 10). Moreover, due to the low number of biologic-naïve patients (*n* = 6), it is not possible to draw any conclusions on the comparison between the different groups. While the study was also limited by missing data, a clear study strength included the assessment of long-term experience with abatacept in routine clinical practice in the Belgian cohort of the ACTION study.

## Conclusion

The Belgian cohort of the ACTION study included RA patients with high disease activity, having failed multiple previous treatment options and presenting with a high comorbidity rate. In this difficult-to-treat RA population, high retention rates and good clinical outcomes were observed with abatacept, consistent with the high retention levels of abatacept IV seen in the overall ACTION study population and in clinical practice. The favorable safety profile of abatacept, as known from the RCT data, was confirmed in routine clinical practice in this RA population. Temporary discontinuation of > 2 consecutive abatacept infusions was not associated with major clinical consequences in this cohort.

## Electronic supplementary material

Below is the link to the electronic supplementary material.

Supplementary file1 (PDF 66 kb)

## Data Availability

BMS policy on data sharing may be found at https://www.bms.com/researchers-and-partners/independent-research/data-sharing-request-process.html.

## Abbreviations

ACTION: AbataCepT In rOutiNe clinical practice
AE: Adverse event
BMI: Body mass index
CCP: Cyclic citrullinated peptide
CD80: Cluster of differentiation 80
CDAI: Clinical disease activity index
CI: Confidence interval
CRP: C-reactive protein
csDMARDs: Conventional synthetic disease-modifying anti-rheumatic drugs
CTLA4: Cytotoxic T-lymphocyte associated protein 4
DAS28: 28-Joint disease activity score
ESR: Erythrocyte sedimentation rate
EULAR: European League Against Rheumatism
HR: Hazard ratio
IV: Intravenous
MTX: Methotrexate
RA: Rheumatoid arthritis
RCT: Randomized clinical trial
SC: Subcutaneous
SD: Standard deviation
SDAI: Simplified disease activity index
SE: Standard error
TNF: Tumor necrosis factor
