# Supplementary material for: Up to 5-year retention of abatacept in Belgian patients with moderate-to-severe rheumatoid arthritis: a sub-analysis of the international, observational ACTION study
Source: Rheumatol Int. 2020 Jun 17;40(9):1409–21. doi: 10.1007/s00296-020-04619-z (PMC7371673; doi:10.1007/s00296-020-04619-z)
Supplement: Supplementary file 1 — Supplementary file1 (PDF 66 kb) [file 296_2020_4619_MOESM1_ESM.pdf]

Up to 5-year retention of abatacept in Belgian patients with moderate-to-severe rheumatoid arthritis: prospective data from a real-world study

Supplementary material

Rheumatology International

R Westhovens[a], SE Connolly[b], J Margaux[c], M Vanden Berghe[d], M Maertens[e], M Van den Berghe[f], Y Elbez[g], M Chartier[h], F Baeke[h], S Robert[h], M Malaise[i]

[a]University Hospitals Leuven, Department of Development and Regeneration, Skeletal Biology and Engineering Research Center Leuven, Belgium

[b]Bristol-Myers Squibb, Princeton, New Jersey, United States

[c]Rheumatology and Physical Medicine Department, Erasme Hospital, Brussels, Belgium.

[d]Grand Hôpital de Charleroi, Charleroi, Belgium

[e]AZ Damiaan, Oostende, Belgium

[f]ASZ Aalst, Wetteren, Belgium

[g]Excelya, Boulogne-Billancourt, France

[h]Bristol-Myers Squibb, Braine-l'Alleud, Belgium

[i]CHU Sart Tilman, Liège, Belgium

Corresponding author

R Westhovens, University Hospitals, Herestraat 49, 3000 Leuven, Belgium. E-mail: rene.westhovens@uzleuven.be (ORCID: https://orcid.org/0000-0002-3432-3073)

Supplementary Table S1: Subgroup analysis-comparison of baseline characteristics of subgroup of patients discontinuing due to lack of efficacy versus the rest of the study population

	Subgroup discontinuation due to lack of efficacy (N=37)	Rest of the study population (N=98)	
**Characteristics**	**Value**	**Value**	**p-value**
**ESR (mL), mean***	32.54	22.27	0.047
**CRP (mg/L), mean****	17.2	8.78	0.0472
**Duration RA, n (%)**			
**≤2 years**	11 (29.3)	9 (9.8)	0.0339
**2–5 years**	9 (24.3)	20 (21.7)	
**5–10 years**	8 (21.6)	26 (28.3)	
**>10 years**	9 (24.3)	37 (40.2)	
**Number of prior csDMARDs, mean (SD)**	2.49 (0.99)	2.02 (1.05)	0.02
**Number of prior anti-TNFs, n (%)**			
**<2**	28 (75.68)	55 (56.12)	0.0474
**≥2**	9 (24.32)	43 (43.88)	

CRP, C-reactive protein; csDMARD, conventional synthetic disease-modifying anti-rheumatic drug; ESR, erythrocyte sedimentation rate; N, number of patients for which the results are known by the physician; n (%), number (percentage) of patients in each category; RA, rheumatoid arthritis; SD, standard deviation; TNF, tumor necrosis factor.

* Number of patients with evaluable data: N=26 for subgroup discontinuing and N=74 for rest of the population.

** Number of patients with evaluable data: N=25 for subgroup discontinuing and N=86 for rest of the population.
